# Neuregulin modulates hormone receptor levels in breast cancer through concerted action on multiple signaling pathways

**DOI:** 10.1042/CS20220472

**Published:** 2022-12-23

**Authors:** Sheila Almaraz Postigo, Juan Carlos Montero

**Affiliations:** 1 Institute of Biomedical Research of Salamanca (IBSAL), Instituto de Biología Molecular y Celular del Cáncer (CSIC-Universidad de Salamanca) and CIBERONC, Salamanca, Spain; 2 Department of Pathology and IBSAL, University Hospital of Salamanca, University of Salamanca, 37007 Salamanca, Spain

**Keywords:** Anti-estrogen therapy, Breast cancer, Estrogen receptor, Neuregulin

## Abstract

The Neuregulins (NRGs) are growth factors that bind and activate ErbB/HER receptor tyrosine kinases. Some reports have described an interplay between this ligand–receptor system and hormonal receptors in breast cancer. However, the mechanisms by which NRGs regulate hormonal receptor signaling have not been sufficiently described. Here, we show that in breast cancer cells the activation of NRG receptors down-regulated ERα through a double mechanism that included post-transcriptional and transcriptional effects. This regulation required the concerted participation of three signaling routes: the PI3K/AKT/mTOR, ERK1/2, and ERK5 pathways. Moreover, these three routes were also involved in the phosphorylation of ERα at serines 118 and 167, two residues implicated in resistance to endocrine therapies. On the other hand, NRGs conferred resistance to fulvestrant in breast cancer cells and this resistance could be reversed when the three pathways activated by NRGs were simultaneously inhibited. Our results indicate that estrogen receptor-positive (ER+) breast tumors that can have access to NRGs may be resistant to fulvestrant. This resistance could be overcome if strategies to target the three main pathways involved in the interplay between NRG receptors and ERα could be developed.

## Introduction

Breast cancer is the most frequently diagnosed cancer in women and represents the fifth leading cause of cancer-related deaths worldwide [1]. About 80% of all breast cancers are positive for hormonal (estrogen or progesterone) receptors [2]. Several therapeutic options for the targeting of those tumors are available. A group of compounds act by binding to the estrogen receptor (ER), preventing its ligand binding (tamoxifen). A second set of drugs can provoke degradation of ER (fulvestrant). Finally, a third type of drugs act by decreasing the levels of estradiol by blocking its biosynthesis (aromatase inhibitors) [3]. Despite these treatments, endocrine resistance occurs due to both intrinsic and acquired resistance [4].

Multiple mechanisms that induce resistance have been described such as mutations or loss of expression of ER, or altered expression of ER pathway genes [4,5]. Another resistance mechanism occurs when the ER is activated independently of its ligand [3]. That type of activation may be due to post-translational modifications such as phosphorylation [3,6]. In fact, overexpression of tyrosine kinase receptors, such as HER2, EGFR, FGFR3, or IGF1R, are capable of phosphorylating the ER, making cells resistant to tamoxifen [5,7–9]. Moreover, the activation of HER2 and HER3 receptors in two resistant MCF7-derived breast cancer cell lines produces endocrine resistance [10]. Interestingly, in one of clones that was resistant to fulvestrant increased expression of the HER receptor ligands Neuregulin 2 and Neuregulin 3 were found [10].

The Neuregulins (NRGs) are a group of polypeptide growth factors that belong to the epidermal growth factor (EGF) family [11]. Four NRG genes (*NRG1–4*) have been identified in humans, which by alternative splicing of the mRNA may produce more than 30 different NRG isoforms. In breast cancer, NRG expression has been linked to response to the anti-HER2 antibody trastuzumab [12]. Moreover, in breast cancer fibroblasts, NRG-induced activation of HER receptors produced resistance to trastuzumab [13]. Interaction of NRGs with HER receptors causes the activation of several intracellular signaling pathways such as the ERK1/2, ERK5, or PI3K/AKT/mTOR routes that regulate different biological processes such as proliferation, migration, and invasion [14,15]. Interestingly, several studies have reported that the activation of MAPKs or the PI3K/AKT pathways is associated with endocrine resistance in breast cancer [5,16,17]. In fact, multiple evidences have indicated that NRGs can induce hormone-independent proliferation in ER+ breast cancer cells [18]. Due to this, it has been proposed that the activation of NRG receptors can induce resistance to endocrine therapy in breast cancer [18–21].

One of the potential mechanisms by which activation of HER2 and HER3 may cause antihormonal therapy resistance is their effect on ER receptors [19,22,23]. Because of clinical relevance of this phenomenon, we decided to gain insights into the mechanisms responsible for the regulation of ER levels by those NRG receptors, and whether such regulation could be responsible for resistance to antihormonal therapy. In the present study, we show that NRG down-regulates ERα at the post-transcriptional and transcriptional levels through the concerted simultaneous activation of several signalling pathways. Moreover, we show that activation of NRG receptors induces ERα phosphorylation, as well as resistance to fulvestrant through the co-operative activation of those signaling pathways.

## Methods

### Reagents and antibodies

Cell culture medium (Dulbecco’s modified Eagle medium [DMEM]), serum (fetal bovine serum [FBS]), penicillin, and streptomycin were purchased from Invitrogen (Gaithersburg, MD). Protein-A Sepharose, MG132, 3-(4,5-dimethylthiazol-2-yl)-2,5-diphenyltetrazolium bromide (MTT), doxycycline, and fulvestrant were from Sigma-Aldrich (St. Louis, MO, U.S.A.). Immobilon®-P transfer membrane was from Merck Millipore Corp. (Darmstadt, Germany). Other generic chemicals were purchased from Sigma, USB Corporation (Cleveland, OH, U.S.A.), Roche Molecular Systems (Madrid, Spain), or Merck (Darmstadt, Germany). Human recombinant Neuregulin-1 β2 was from Prospec Protein Specialists (Rehovot, Israel). The dual inhibitor of PI3K and mTOR, BEZ235 and the mTOR inhibitor, rapamycin were from LC Laboratories (Woburn, MA, U.S.A.). The MEK1/2 inhibitors, AZD6244 and trametinib, the MEK5 inhibitor, BIX02189, and the PI3Kα inhibitor, Alpelisib were from Selleckchem (Houston, TX, U.S.A.). The ERK5 inhibitor JWG071 was from Glixx Laboratories Inc (Hopkinton, MA, U.S.A.). The RNA extraction kit was from Qiagen (Hilden, Germany). Primers for qPCR, M-MLV reverse transcriptase, and oligo-dT were from Invitrogen (Gaithersburg, MD). The SYBR Select Master Mix for CFX and the Quant Studio 1 System to run the qPCR were from Applied Biosystems (ThermoFisher Scientific, Waltham, MA, U.S.A.). The anti-ERα and anti-PR antibodies used for western blot were from Master Vitro Diagnostica (Granada, Spain). The anti-HER2 (Ab3) and the anti-tubulin antibodies were from Calbiochem (La Jolla, CA, U.S.A.). The antiphospho-Tyr (PY99), anti-ERK1/2, and anti-calnexin were from Santa Cruz Biotechnology (Santa Cruz, CA, U.S.A.). Antibodies for phospho-AKT (Ser473), phospho-ERK1/2 (Thr202/Tyr204), S6, phospho-S6 (Ser240/244), phospho-ERα (Ser118), and phosphor-ERα (Ser167) were from Cell Signaling Technologies (Beverly, MA, U.S.A.). The anti-AKT antibody was from BD Biosciences (San Jose, CA, U.S.A.). Antibodies for HER3, NRG, and phospho-ERK5 were produced in our laboratory [15,24,25]. The horseradish peroxidase (HRP)-conjugated antibodies: anti-mouse, anti-rabbit, and anti-rabbit light-chain specific were obtained from GE Healthcare Life Sciences (Piscataway, NJ, U.S.A.), Bio-Rad Laboratories (Hercules, CA, U.S.A.), and Jackson ImmunoResearch Laboratories (West Grove, PA, U.S.A.), respectively.

### Cell culture

Six breast cancer cell lines—MCF7, BT474, T47D, SKBR3, MDA-MB231, and HS578T—were grown in DMEM supplemented with 10% FBS, containing high glucose (4500 mg/l) and antibiotics (100 mU/ml penicillin, 100 μg/ml streptomycin). Cell lines were cultured at 37°C in a humidified atmosphere in the presence of 5% CO_2_ and 95% air. The cell lines were obtained from the American Type Culture Collection Cell Biology Collection (Manassas, VA, U.S.A.). The MCF7 cell line in which proNRGα2c was expressed in a regulated manner using the tetracycline transactivator system (MCF7-Tet off-NRGα2c) has been described [26]. Where indicated, cells at 80% confluence were serum-starved for 16–18 h and pretreated with the different drugs at the concentrations indicated in the figure legends. Subsequently, cells were stimulated or not with 10 nM NRG for the indicated times and collected for protein extraction. For MCF7-Tet off-NRGα2c experiments, cells were treated with doxycycline for 72 h to eliminate the expressed NRG.

### Protein extraction, immunoprecipitation, and western blotting

Cells were washed with phosphate-buffered saline and lysed in ice-cold lysis buffer (140 mM NaCl, 50 mM ethylenediaminetetraacetic acid, 10% glycerol, 1% Nonidet P-40, 20 mM Tris, pH 7.0, 1 μM pepstatin, 1 μg/ml aprotinin, 1 μg/ml leupeptin, 1 mM phenylmethyl sulphonyl fluoride (PMSF), 25 mM β-glycerophosphate, 50 mM sodium fluoride, and 1 mM sodium orthovanadate). Samples were centrifuged at 10000 ***g*** at 4°C for 10 min, and supernatants were transferred to new tubes. For immunoprecipitation, samples were incubated with the corresponding antibody and protein A-Sepharose. Immunoprecipitations were performed at 4°C for 2 h, and immune complexes were recovered by a short centrifugation, followed by three washes with 1 ml of cold lysis buffer. Samples were then boiled in 2× electrophoresis sample buffer, and resolved in 6–15% SDS-PAGE gels, depending on the molecular weight of the proteins to be analyzed. After electrophoresis and transfer to Immobilon-P membranes, the filters were blocked in Tris-buffered saline with Tween (TBST) (100 mM Tris [pH 7.5], 150 mM NaCl, 0.05% Tween 20) containing 1% of bovine serum albumin for 1 h and then incubated with the corresponding antibody for 2–16 h. After washing with TBST, membranes were incubated with HRP-conjugated anti-mouse or anti-rabbit secondary antibodies for 30 min and bands were visualized by using the ChemiDoc Detection System (Hercules, CA, U.S.A.). Densitometric measurements of the bands were performed using the Image LabTM Software Version 6.0.1 Bio-Rad Laboratories (Hercules, CA, U.S.A.). Different loading controls were used (calnexin or tubulin), depending on the molecular weight of the proteins of interest and the gel percentage.

### Quantitative retrotranscriptase-PCR

Total RNA from MCF7, BT474, and T47D cells was isolated using the RNeasy Kit according to the manufacturer’s instructions. First-strand cDNA was synthesized using M-MLV reverse transcriptase and oligo-dT following the instructions from the manufacturer (Invitrogen, Carlsbad, CA, U.S.A.). Quantitative retrotranscriptase-PCR (qRT-PCR) assays were performed in duplicate in 96-well optical plates on a Quant Studio 1 System with SYBR Select Master Mix for CFX. Levels of ERα and PR mRNA were normalized against that of GAPDH and were relativized to those from unstimulated cells using the 2(ΔΔCt) method. The sequences of the primers used for gene expression analysis were: ERα forward 5′-TTGGCCAGTACCAATGACAA-3′; ERα reverse 5′-CAATGGTGCACTGGTTGGT-3′; PR-AB forward 5′-CGCGTTCCTACCTTGTGG-3′; PR-AB reverse 5′-CCTCCGCTTTGTACAGGATG-3′; GAPDH forward 5′-GAGTCAACGGATTTGGTCGT-3′, and GAPDH reverse 5′-GATCTCGCTCCTGGAAGATG-3′.

### Cell proliferation assays

Cell proliferation experiments were performed using MTT assays, where MTT is reduced to purple formazan by the mitochondria of living cells. Increased or decreased in cell number is detected by MTT metabolization. The cells were plated at a density ranging from 6000 cells to 20000 cells per well in 24-well plates and cultured overnight in DMEM supplemented with 10% FBS. The next day (day 1 of culture), the medium was replaced with DMEM without FBS or with 1% FBS and the cells were treated with the corresponding agents depending on the experiment. Cell proliferation was analyzed at 4 days. Each well was replaced with 250 μl of fresh medium containing MTT (0.5 μg/μl) and incubated at 37°C for 1 h. The medium was then eliminated and 500 μl of dimethyl sulfoxide was added to each well. The plate was agitated in the dark during 10 min to dissolve the MTT-formazan crystals. The absorbance of the samples was measured at 570 nm in a multiwell plate reader (Tecan ULTRA Evolution, Männedorf, Switzerland). The reference value was made 24 h after the cells were plated and before the addition of the stimuli or drugs. Results were plotted as the mean ± SD values of quadruplicates from a representative experiment that was repeated at least two independent times.

Cell proliferation assays were also performed by cell counting. The cells were plated at a density of 10000 cells for MCF7 and 60000 cells for BT474 per well in 6-well plates and cultured overnight in DMEM supplemented with 10% FBS. Twenty-four hours later, the medium was replaced with DMEM with 1% FBS and the cells were stimulated with NRG (10 nM) for 24 h, and treated with Fulvestrant (1 μM). Cell proliferation was analyzed at 4 days. Cells were collected and counted using a Z1 Coulter Particle Counter (Beckman Coulter, Pasadena, CA, U.S.A.). Results were plotted as the mean ± SD values of triplicates from a representative experiment that was repeated at least two times.

### Statistical analyses

Data were analyzed statistically using the software package SPSS 26.0 (SPSS Inc., Chicago, IL, U.S.A.). Comparison of continuous variables between two groups in the experiments were performed using a Mann–Whitney U-test. Differences were considered statistically significant when the *P-*value was less than 0.05. All experiments were repeated at least twice.

## Results

### NRG decreases ERα levels at translational and transcriptional levels

**Figure 1:**
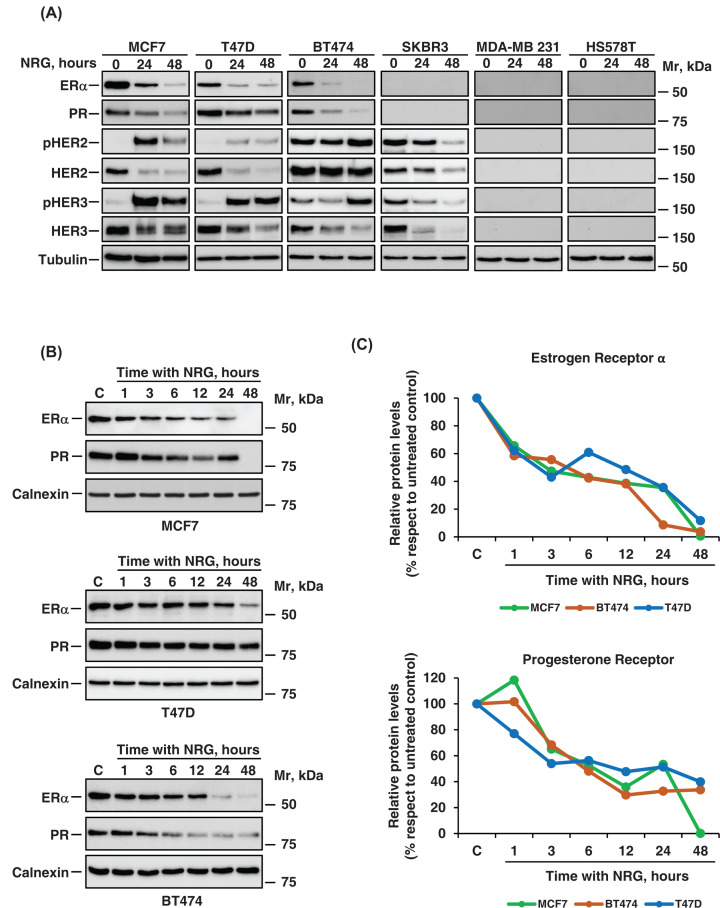
NRG down-regulates ERα and PR protein levels in breast cancer (**A**) Western blotting analysis of the effect of NRG in different breast cancer cell lines. For these experiments, we used hormone receptor-positive (MCF7, T47D); HER2-positive (BT474, SKBR3), and triple-negative (MDA-MB231, HS578T) cell lines. Cells were treated with NRG (10 nM) at the indicated times, and then the levels of ERα, PR, HER2, HER3, p-HER2, and p-HER3 were analyzed by western blot. Tubulin was used as a loading control. The position of the Molecular rate (Mr) markers is shown at the right. (**B**) Time-course effect of NRG on ERα and PR levels. MCF7, T47D, and BT474 cells were treated with NRG for the indicated times. Lysates were analyzed by western blot with anti-ERα and anti-PR antibodies. Calnexin was used as a loading control. (**C**) Quantitation of the levels of ERα and PR from the experiment performed in (**B**). The graphs represent the relative protein levels (arbitrary units) of ERα or PR respect to untreated control (0 h).

ERα and progesterone (PR) expression was first analyzed by Western in six different cell lines representative of hormone receptor positive (MCF7, T47D), HER2 positive (SKBR3, BT474), and triple-negative (MDA-MB231, HS578T) breast cancer subtypes. Both hormonal receptors were constitutively expressed in MCF7, T47D, and BT474 cells (Figure 1A). The latter cell line, while being prototypically used to study HER2+ tumors, has also been reported to express hormone receptors and has been considered by some authors as representative of the luminal B subtype [27]. ERα and PR were undetectable in SKBR3, MDA-MB231, or HS578T cell lines. The NRG receptors HER2 and HER3 were present in MCF7, T47D, BT474, and SKBR3 cells, but not in the triple-negative cell lines. As shown in Figure 1A, treatment with NRG decreased the levels of ERα and PR at 24 and 48 h in MCF7, T47D, and BT474 cells. More detailed temporal analyses showed that the *t*_1/2_ of the effect of NRG on the levels of ERα and PR was between 3–6 h of treatment with the growth factor (Figure 1B,C). Moreover, a slight increase in PR levels was observed in the MCF7 and BT474 cell lines in the first hour of stimulation with NRG (Figure 1B,C).

**Figure 2:**
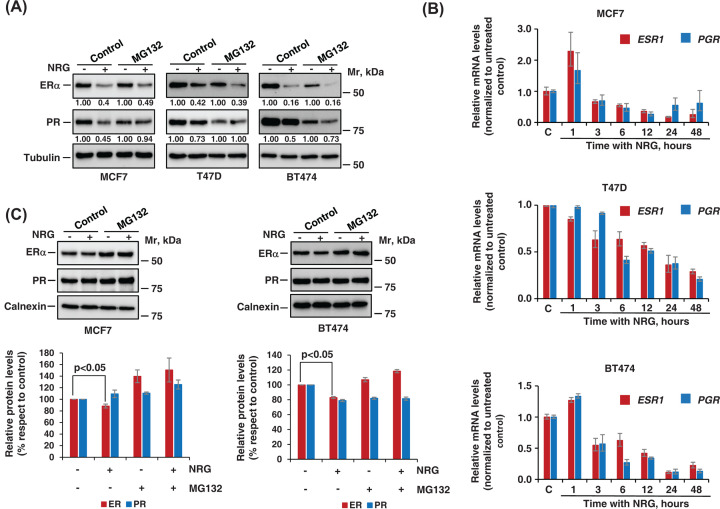
NRG decreases ERα levels at translational and transcriptional levels (**A**) Role of the proteasome in the regulation of ERα levels by NRG receptors. MCF7, T47D, and BT474 cells pretreated with the proteasome inhibitor MG132 (5 μM) for 1 h were stimulated with NRG during 24 h and lysed. The levels of ERα and PR were analyzed by western blot. Tubulin was used as a loading control. The numbers shown below the blots indicate quantitative measurements of ERα and PR relative to their unstimulated control. (**B**) Quantitative RT-PCR detection of *ESR1* (estrogen receptor alpha gene) and *PGR* (progesterone receptor gene) mRNA levels in NRG-stimulated MCF7, T47D, and BT474 cells. *ESR1* and *PGR* mRNA levels were normalized against that of GAPDH and relativized to unstimulated control cells. The graph represents the mean ± SD of data from three independent experiments. The significance *P*-values are shown in Supplementary Table S1. (**C**) NRG induces the degradation of ERα via proteasome at short times. MCF7 and BT474 cells were pretreated with or without MG132 (10 μM) for 1 h and then stimulated with NRG during 2 h. The levels of ERα and PR were analyzed by western blotting. Calnexin is shown as a loading control. The graphics represent the quantitation of ERα and PR levels in MCF7 and BT474 cell lines corresponding to the mean ± SD of data from two independent experiments.

The decrease in ERα and PR protein levels caused by NRG may be due to an increase in their degradation, a decrease in their synthesis or both. To analyze this, we first inhibited the proteasome using the drug MG132 during 1 h and later we analyzed the levels of ERα and PR after stimulation with NRG during 24 h. Former experiments established that this incubation time with NRG was sufficient to observe a substantial decrease in ERα and PR at the protein level. In none of the three lines analyzed the proteasome inhibitor was able to substantially prevent the decrease in ERα caused by activation of the NRG receptors (Figure 2A). However, in the case of PR, MG132 prevented the decrease in PR induced by NRG in MCF7, T47D, and BT474 cells. These studies suggested that degradation via proteasome at long times is not the only mechanism for the decrease in ERα and PR after stimulation with NRG. Therefore, whether the decrease in ERα and PR by NRG were due to transcriptional regulation was explored. Levels of ERα, and PR mRNA decreased during NRG treatment (Figure 2B and Supplementary Table S1). As occurred in the protein experiments, the mRNA levels of PR in the MCF7 and BT474 cell lines also increased in the first hour of treatment. After 3 h of treatment, the levels of PR decreased with respect to those present in the untreated control. Of note, in MCF7 and BT474 cell lines, an increase in the mRNA in the first hour of treatment was also observed for the ERα (Figure 2B); however, this did not occur at the protein level (Figure 1B,C). Due to this, we decided to investigate whether at short times of treatment with NRG (1–2 h), the decrease in protein levels of ERα were regulated via proteasome. The protein levels of ERα were analyzed in MCF7 and BT474 cells previously pretreated with the proteasome inhibitor, and later stimulated with NRG for 2 h. Two-hour treatment with NRG produced a slight but significant reduction in ERα levels, which was prevented by proteasome inhibitor (Figure 2C). Together, these experiments indicated that the decrease in ERα and PR were provoked by transcriptional as well as post-transcriptional mechanisms.

### NRG controls ERα and PR levels through several signaling routes

**Figure 3:**
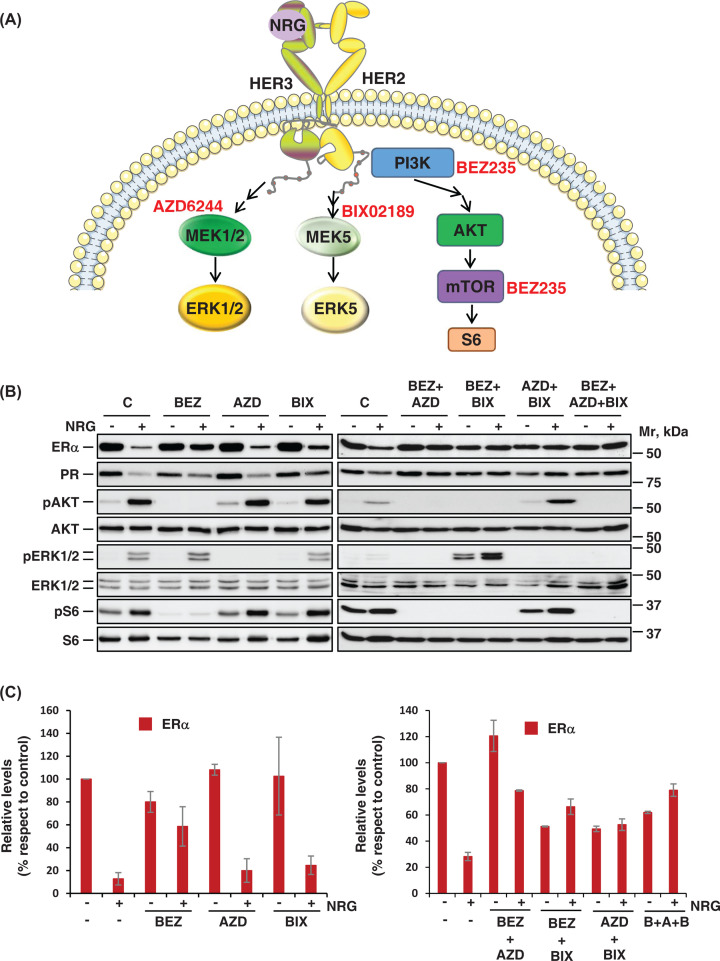
NRG controls ERα and PR levels through several signaling routes (**A**) Schematic representation of the signaling pathways that are activated by NRG-ErbB receptors. Inhibitors used to block the different signaling pathways are shown in red. (**B**) Analysis of the effect of different signaling pathway inhibitors on the down-regulation of ERα and PR induced by NRG in MCF7 cells. MCF7 cells were pretreated with a PI3K inhibitor (BEZ235, 1 μM), a MEK1/2 inhibitor (AZD6244, 5 μM), a MEK5 inhibitor (BIX02189, 10 μM) and with double and triple combinations of these inhibitors for 2 h. Later, they were then stimulated with NRG for 24 h. The expression levels of ERα, PR, and several of the downstream proteins involved in NRG receptor signaling were performed by western blot. (**C**) The graphic represents the quantitation of ERα levels corresponding to the mean ± SD of data from three independent experiments as performed in (**B**).

Stimulation of ErbB/HER receptors by NRG causes the activation of various intracellular signaling routes such as the ERK1/2, PI3K/AKT/mTOR, and ERK5 pathways. To explore the contribution of these routes to NRG-induced ERα and PR decrease, drugs that target different components of these signaling pathways were used (Figure 3A). In agreement with previously published data, western blotting experiments confirmed that NRG activated phosphorylation of S6 and AKT, which are used as readouts of activation of the PI3K/mTOR route. On the other hand, NRG also activated the ERK1/2 and ERK5 route, as indicated by the dual phosphorylation of ERK1/2 (Figure 3B and Supplementary Figure S1) and the phosphorylation of ERK5 (data not shown and Figure 5E) in their TEY activation microdomain. Inhibitors targeting upstream components of these three routes such as BEZ235, a dual PI3K/mTOR inhibitor, AZD6244, a highly selective inhibitor of the ERK1/2 upstream-activating kinases MEK1/2, and BIX02189, an inhibitor of MEK5, which is the ERK5 upstream-activating kinase [28] were tested (Figure 3A). Preincubation with BEZ235 prevented NRG-induced phosphorylation of AKT and S6, without affecting the effect of NRG on ERK1/2 and ERK5 (Figures 3B and 5E and Supplementary Figure S1). On the other hand, AZD6244 and BIX02189 inhibited the capability of NRG to provoke increases in the phosphorylation of ERK1/2 and ERK5, respectively (Figures 3B and 5E and Supplementary Figure S1). When added individually, these drugs affected the action of NRG on ERα and PR although in a different way depending on the cell type (Figure 3B and Supplementary Figure 1). Thus, in MCF7 cells, BEZ235 was the most efficient drug in causing down-regulation of ERα induced by NRG (Figure 3B,C). However, in BT474 cells, AZD6244 was the drug that mostly affected the down-regulation of ERα (Supplementary Figure S1C,D). Importantly, the efficacy of these drugs to prevent NRG-induced down-regulation of ERα and PR increased when they were combined. Thus, while in general the double combinations were clearly superior to the individual treatments, the most notable effect was obtained by the triple combination. In fact, treatment with BEZ235, AZD6244, and BIX02189 completely abrogated NRG-induced ERα and PR decrease in all three cell lines analyzed (Figure 3B,C, and Supplementary Figure S1). Other inhibitors that block these pathways (PI3K-mTOR, ERK1/2, and ERK5) produced similar results (Supplementary Figure S1E,F).

**Figure 4:**
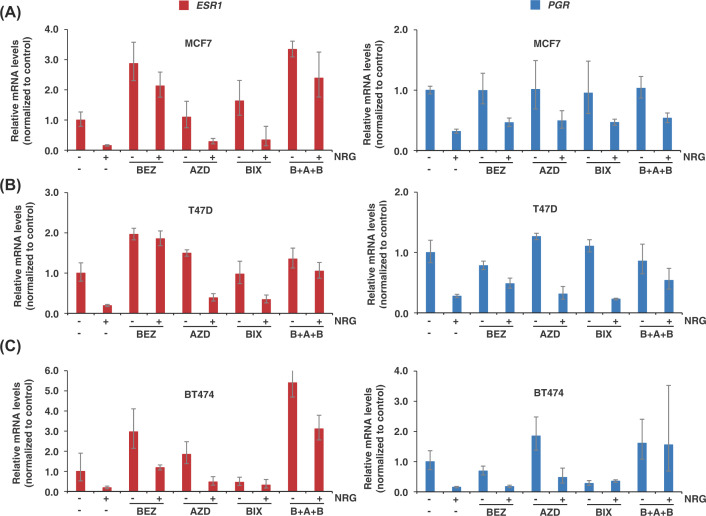
Several pathways activated by NRG receptors control the *ESR1* and *PGR* mRNA levels Graphical representation of the relative mRNA levels of *ESR1* and *PGR* in MCF7 (**A**), T47D (**B**), and BT474 (**C**) cells pretreated with the indicated inhibitors and later stimulated with NRG for 24 h. The graph represents the mean ± SD of data from two independent experiments. The significance *P*-values are shown in Supplementary Table S2.

At the transcriptional level, the three inhibitors used individually substantially prevented the decrease in ERα mRNA upon NRG stimulation compared with the control sample in the three cell lines used (Figure 4A–C). It should be noted that BEZ235 was the most efficient in preventing the effect of NRG on ERα receptor mRNA levels. With respect to the PR, the inhibitors used individually slightly prevented the decrease in PR mRNA levels upon NRG stimulation compared with the control. On the other hand, the triple combination was clearly superior in blocking the decrease in PR mRNA levels after stimulation with NRG in the BT474 cell line; however, this blocking was very discrete in MCF7 and T47D cell lines.

### Multiple signaling pathways control ERα S^118^ and S^167^ phosphorylation by NRG

**Figure 5:**
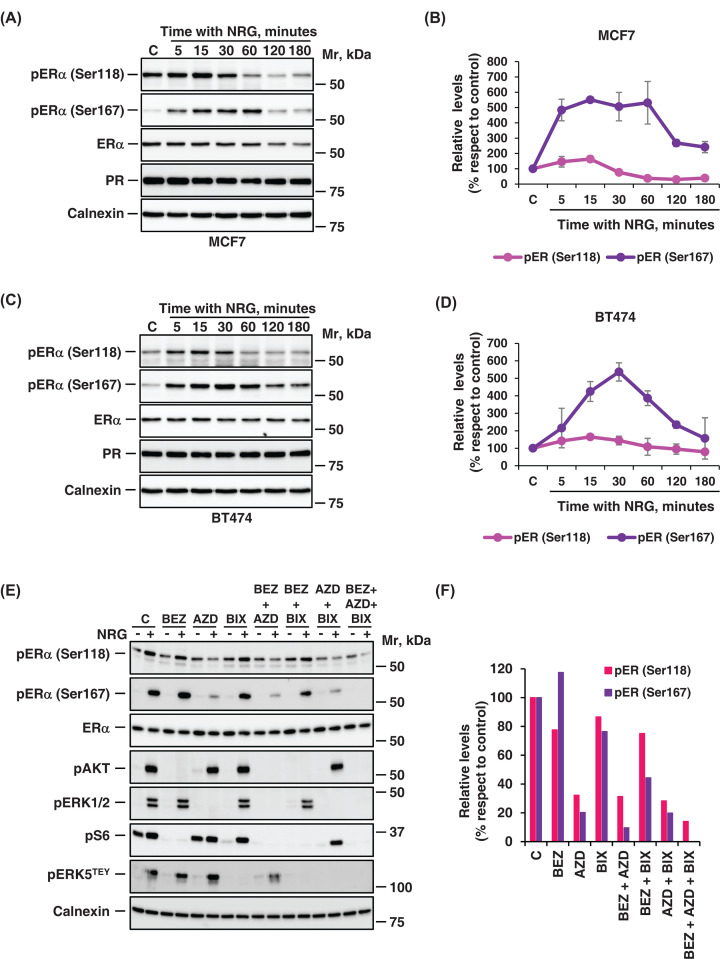
NRG controls phosphorylation of ERα at Serine 118 and 167 through several signaling routes (**A, C**) Kinetics of serine phosphorylation of ERα in response to NRG. MCF7 (**A**) or BT474 (**C**) cells were stimulated with NRG at indicated times and lysed. The levels of phosphorylation of ERα at serine 118 and 167 and total levels of ERα and PR were analyzed by western blot. (**B, D**) The graphics represents the quantitation of the phosphorylation of the different serine residues (S118 and S167) phosphorylated in ERα, corresponding tothe mean ± SD of data from two independent experiments as performed in (**A**) and (**C**). (**E, F**) The levels of phosphorylation of ERα at serine 118 and 167 in MCF7 cells pretreated with inhibitors (BEZ, AZD, and BIX) alone or in combination were analyzed. The effect of these inhibitors was verified by analyzing the different downstream proteins of these routes. In (**A**, **C**, and **E**), calnexin was used as a loading control.

Since NRG-induced down-regulation of ERα appeared to be controlled through multiple intracellular signaling pathways, we decided to investigate whether these pathways could regulate the phosphorylation of ERα at serine 118 and 167. Phosphorylation of the ERα at those two residues has been reported to be relevant for the control of several functions of ERα such as DNA binding, transcription, co-activator binding or protein stability [29]. Moreover, the phosphorylation at those residues has also been implicated in resistance to endocrine therapies [29–33]. Time course experiments showed that treatment of MCF7 or BT474 cells with NRG induced the phosphorylation of ERα in serines 118 and 167 (Figure 5A,C). Quantitation of the phosphorylation levels of these residues showed that the maximum peak of phosphorylation occurred between 15 and 60 min after treatment with NRG in MCF7 and BT474 cells, respectively (Figure 5B,D). On the other hand, phosphorylation in serine 167 was sustained longer than phosphorylation in serine 118 (Figure 5A–D). In the two lines analyzed the phosphorylation of serine 118 in untreated cells (in basal conditions) was higher than serine 167 (Figure 5A–D).

The contribution of the different NRG-activated signaling pathways on ERα phosphorylation at these residues was analyzed using the specific inhibitors that block the three main routes (ERK1/2, PI3K/AKT/mTOR, and ERK5 pathways). Only the MEK1/2 inhibitor AZD6224 was able to substantially prevent NRG-induced serine 118 phosphorylation (Figure 5E,F). Moreover, the triple combination of inhibitors was slightly more efficient in preventing phosphorylation at this residue, compared with AZD6224 individually (Figure 5E,F). On the other hand, NRG-induced S167 phosphorylation was drastically reduced by AZD6224 and slightly by BIX02189. Moreover, the double combinations of BEZ235 plus AZD6224 was able to slightly decrease the phosphorylation of this residue more than the individual inhibitors (Figure 5E,F). The triple combination of inhibitors (AZD6224, BIX02189, and BEZ235) completely inhibited NRG-induced S167-ERα phosphorylation (Figure 5E,F).

### Modulation of fulvestrant action by NRG involves multiple signalling pathways

**Figure 6:**
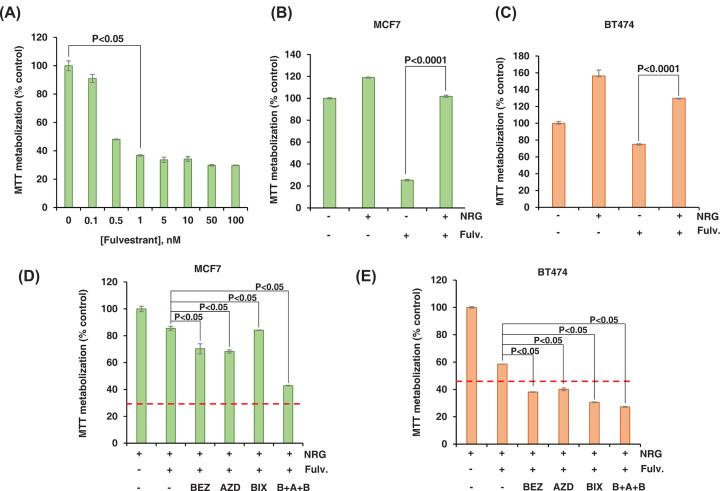
NRG induces resistance to fulvestrant through several signaling routes in breast cancer cell lines (**A**) Dose-response analyses of the effect of fulvestrant on MCF7 cells. Cells were treated with fulvestrant at the indicated doses and the cell proliferation was determined by MTT metabolization 4 days later. The data are plotted as the percentage of MTT metabolization with respect to control. Results are shown as the mean ± SD of duplicates of an experiment repeated three times. (**B, C**) Effect of NRG in MCF7 or BT474 cells treated with fulvestrant. MCF7 (**B**) or BT474 (**C**) cells were stimulated with NRG (10 nM) for 24 h, and later the cells were treated with Fulvestrant (1 μM). Cell proliferation was determined by MTT metabolization 4 days later. The data are plotted as the percentage of MTT metabolization with respect to control. Results are shown as the mean ± SD of quadruplicates of an experiment repeated two times. (**D, E**) Effect of several signaling pathway inhibitors on NRG-induced resistance to fulvestrant. MCF7 (**D**) or BT474 (**E**) cells were plated in DMEM with 10% FBS at a density of 6000 (MCF7) or 12000 (BT474) cells per well in a 24-multiwell plate. The next day, the medium was replaced with DMEM with 1% FBS containing BEZ235 (1 nM) or AZD6244 (500 nM) or BIX02189 (1μM) or the triple combination of these compounds for 2 h. Then, the cells were stimulated with NRG (10 nM) during 24 h and treated with fulvestrant (1 nM) and cell proliferation was determined by MTT metabolization 4 days later. The red dashed line indicates the percentage of MTT metabolization of fulvestrant-treated samples with respect to the control. Results are shown as the mean ± SD of duplicates of an experiment repeated three times.

As NRG increased the phosphorylation of the ERα in two residues (serine 118 and 167) that have been involved in resistance to endocrine therapies and also decreased ERα expression levels through the activation of multiple signaling pathways that have been implicated in endocrine resistance, we decided to investigate whether NRG could also confer resistance to antiestrogen therapies. In MCF7 cells, fulvestrant was able to inhibit cell proliferation in a dose-dependent manner, reaching a maximal effect at 50 nM and with an IC_50_ value of 0.459 nM (Figure 6A). However, the effect of fulvestrant in BT474 and T47D cell lines was less pronounced, requiring 500 nM to achieve a saturating effect (Supplementary Figure S2A). As fulvestrant causes ERα degradation and the stimulation of MCF7 cell with NRG also decreases ERα levels, we explored in these cells the effect of adding fulvestrant plus NRG on ERα levels. As shown in Supplementary Figure S2B, the addition of fulvestrant to MCF7 cells treated with NRG decreased ERα levels more than the individual treatments. To explore whether NRG confers resistance to fulvestrant, MCF7 and BT474 cells were treated with NRG for 24 h and then fulvestrant was added. The proliferation of these cells was analyzed by MTT metabolization (Figure 6B,C) or cell counting (Supplementary Figure S3A,B). As seen in these figures, treatment with NRG conferred resistance to the treatment with fulvestrant. Reversing the order of addition of the drug (Fulvestrant treatment prior to NRG) generated similar results (Supplementary Figure S3C,D). This result was also similar when we used the MCF7^Tet off^-NRGα2c cell line in which the proNRGα2c is regulated using the tetracycline transactivator system (Supplementary Figure S2C). Those cells were engineered to express proNRGα2c at will [26].

Inhibitors of the ERK1/2, PI3K/AKT/mTOR, and ERK5 pathways were able to partially block NRG-induced resistance to fulvestrant (Figure 6D,E). On the other hand, the triple combination exerted a higher blocking effect on the action of NRG than the individual inhibitors. Together, the above results indicated that NRG may inhibit the action of drugs, such as fulvestrant, that are used to treat hormone receptor-positive tumors.

## Discussion

The cross-talk between the NRG-ErbB and hormonal receptors in breast cancer has already received research attention because of the important roles of these ligand–receptor systems in the pathophysiology of that disease. In fact, several studies have described that the activation of HER receptors by NRG regulates the levels and activity of ERα [19,22,23]. However, the precise mechanisms by which such regulation occurs have not been fully defined. Considering that, we decided to investigate the signaling pathways activated by NRG receptors responsible for the regulation of ERα levels. In the present work, and in addition to the identification of the signaling pathways that NRG receptors use to control the levels of ERα and PR, we demonstrate that such regulation is complex and requires the collaboration of three different signaling routes activated by NRG. We also show that the activation of NRG receptors induced ERα phosphorylation, as well as resistance to fulvestrant through the simultaneous activation of multiple signaling pathways.

Stimulation of ErbB receptors with NRG caused decreased of the levels of ERα and PR in MCF7, T47D, and BT474 cells. The stimulation of ErbB receptors by NRG could be one of the reasons of the nonexpression of hormone receptors in triple-negative breast cancer [34]. However, our unpublished data indicated that triple-negative breast cancer cells that express NRG do not express ErbB3 or ErbB2. Therefore, the absence of ERα and PR in these cells is not due to activation of the NRG1/ErbB3/ErbB2 axis. Quantitative RT-PCR and proteasome inhibitor analyses revealed that the decrease in ERα and PR induced by NRG had a double mechanism of action, namely degradation via proteasome as well as reduction in ERα and PR mRNA levels. With respect to the signaling routes that NRG uses to control those ERα and PR levels, inhibitor studies allowed the identification of the PI3K/AKT/mTOR, the ERK1/2, and the ERK5 routes. In fact, only the simultaneous inhibition of these three pathways totally prevented the decrease in ERα and PR induced upon activation of NRG receptors. Similar results were obtained at the mRNA level for ERα. However, for PR, only the simultaneous inhibition of the three routes was superior in the BT474 cell line. These results show for the first time that NRG regulates the levels of ERα at the protein level and of mRNA through the simultaneous activation of different intracellular signaling pathways.

NRG induced phosphorylation of the ERα in S118 and S167, two residues involved in DNA binding, transcription, co-activator binding, RNA splicing, cell growth, and invasion and protein stability. In fact, estradiol and growth factors such as IGF1 or EGF have been reported to induce the phosphorylation of serine 118 and 167 of the ERα [29]. These residues are located in the AF-1 domain and their phosphorylation has been implicated in the regulation of ERα activity [35–37]. The phosphorylation of these residues by NRG could regulate ERα activity and this could trigger a negative feedback to reduce ERα levels and thus suppress its activity. Experiments using inhibitors of the main pathways activated by NRG receptors revealed that the ERK1/2 pathway was the main route that regulated phosphorylation of residue S118, in agreement with the fact that ERK1/2 is one of the kinases that have been described that phosphorylates this residue in a ligand-independent manner [38,39]. Of note, it has been described that this residue is also phosphorylated by the ERα ligand without the intervention of ERK1/2 [39], due to which, other kinases have been involved in the phosphorylation of ER in S118 [29]. In fact, in our cellular model, the PI3K-AKT-mTOR and ERK5 pathway also contributed to the regulation of phosphorylation of this residue in response to NRG receptor activation. The simultaneous inhibition of the three signal pathways activated by NRG receptors reduced the phosphorylation of ERα in S118 to basal levels. Activation of NRG receptors also induced ERα phosphorylation at S167. Like residue S118, the main route that regulates phosphorylation of ERα in S167 was the ERK1/2 pathway. Interestingly, it has been reported that this residue can be phosphorylated also by AKT [35]. In fact, the other two pathways activated by NRG receptors (PI3K-AKT-mTOR and ERK5) also regulated the phosphorylation of this residue but to a lesser degree. These results suggest that the phosphorylation in these residues is controlled by several kinases that are activated through different signaling pathways.

In MCF7 cells, the activation of ErbB receptors by NRG increased ERα phosphorylation at serine 118 and 167, two residues implicated in resistance to endocrine therapies. In fact, an increase in phosphorylation of serine 118 in ERα has been reported in tamoxifen resistant cell lines [30]. In addition, the activation of RET receptor tyrosine kinase induces phosphorylation in serine 118 in ERα and induces resistance to Tamoxifen [31]. On the other hand, *in-vitro* studies have shown that phosphorylation of ERα at serine 167 reduces sensitivity to tamoxifen [32,33]. Whether phosphorylation of these residues after activation of NRG receptors in MCF7 cells is important in resistance to endocrine therapies, such as fulvestrant, remains to be determined.

The activation of NRG receptors induced the activation of several signaling pathways that have been implicated in endocrine resistance in breast cancer. Stimulation of MCF7 or BT474 cells with NRG resulted in resistance to fulvestrant. This resistance was regulated through the ERK1/2, ERK5, and PI3K/AKT/m-TOR pathways. These data are in agreement with the results obtained by Sandra et al. [10]. These authors generated clones resistant to tamoxifen and fulvestrant in MCF7 cells in which they observed activation of the ERK1/2 and PI3K/AKT signaling pathways. Moreover, the inhibition of both routes reversed the resistance to fulvestrant and tamoxifen. Interestingly, in one of their clones that was resistant to fulvestrant an increased expression of NRG2 and NRG3 was found. These authors concluded that the activation of receptors by these ligands and others produces an increase in the activation of the ERK1/2 and PI3K/AKT pathway and that their inhibition can reverse resistance to endocrine therapies. In our experiments, in addition to these two pathways, we also observed that inhibition of the ERk5 pathway is capable of reversing NRG-induced resistance to fulvestrant. Therefore, in our model, the activation of the three pathways is necessary to generate resistance to fulvestrant induced by NRG.

**Figure 7:**
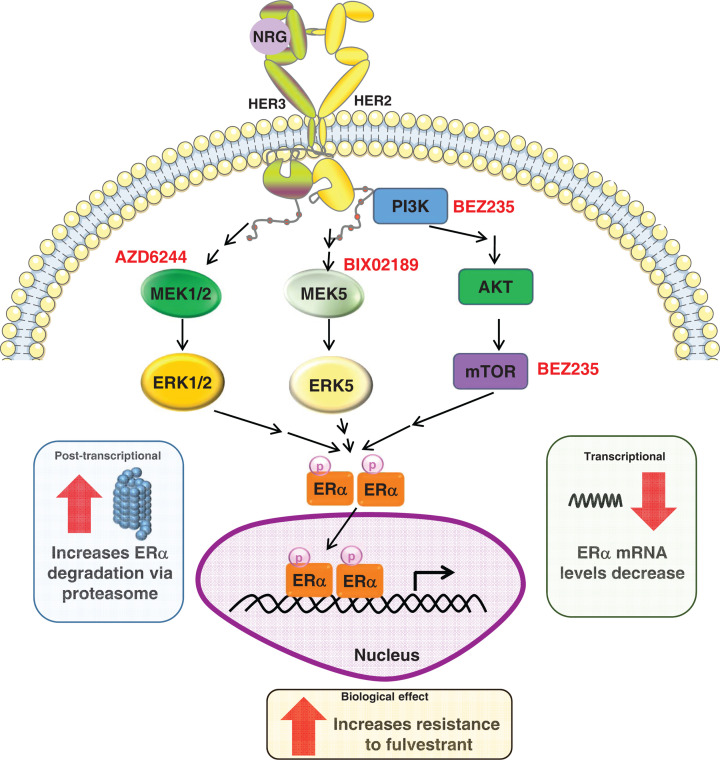
Schematic representation of the effect of NRG on ERα in breast cancer Soluble NRG binds and activates ErbB/HER receptors and triggers the activation of several intracellular signaling pathways (ERK1/2, ERK5, and PI3K-AKT-mTOR). These pathways regulate simultaneously ERα phosphorylation at serines 118 and 167 and their expression levels through the proteasome or at the transcriptional level. Furthermore, simultaneously activation of these signaling pathways by NRG receptors induce resistance to fulvestrant. Inhibitors used to block the different signaling pathways are shown in red.

In conclusion, our results show for the first time that the activation of HER receptors by NRG induces phosphorylation of ERα and decreases their levels through simultaneous activation of PI3K/AKT/mTOR, ERK1/2, and ERK5 pathways. That fact, together with the activation of pro-oncogenic signaling routes by NRG may cause resistance to fulvestrant (Figure 7). Our results suggested that ER+ breast tumors that express NRG or that are fed by NRG may be resistant to fulvestrant. In clinical practice, this resistance could be reversed if these tumors are treated with a combination of inhibitors that block the main pathways (ERK1/2, ERK5, and PI3K-AKT-mTOR) that are activated by NRG receptors.

## Clinical perspectives

- Interaction of NRGs with HER receptors causes the activation of several intracellular signaling routes that regulate different biological processes. Moreover, NRG can induce hormone-independent proliferation in ER+ breast cancer cells. Due to this, it has been proposed that the activation of NRG receptors can induce resistance to endocrine therapy in breast cancer.
- Simultaneous activation of PI3K/AKT/mTOR, ERK1/2, and ERK5 pathways by NRG receptors regulates phosphorylation and levels of ERα and induces resistance to fulvestrant. These results suggest that ER+ breast tumors that have access to NRG may be resistant to fulvestrant.
- In the clinical practice, the resistance to fulvestrant could be overcome if the tumors are treated with a combination of inhibitors that block the main pathways that are activated by NRG receptors. However, combining multiple inhibitors targeting routes essential for cell viability may result in toxicity.

## Supplementary Material

Supplementary Figures S1-S3 and Tables S1-S2

## Data Availability

All the supporting data are included in the main text and supplementary files.

## Abbreviations

DMEM: Dulbecco’s modified Eagle medium
EGF: epidermal growth factor
ER: estrogen receptor
FBS: fetal bovine serum
MAPK: mitogen activated protein kinase
Mr: molecular rate
MTT: 3-(4,5-dimethylthiazol-2-yl)-2,5-diphenyltetrazolium bromide
NRG: neuregulin
PR: progesterone receptor
qRT-PCR: quantitative retrotranscriptase-PCR
TBST: tris-buffered saline with Tween
