# Supplementary material for: Neuregulin modulates hormone receptor levels in breast cancer through concerted action on multiple signaling pathways
Source: Clin Sci (Lond). 2022 Dec 23;137(1):1–15. doi: 10.1042/CS20220472 (PMC9805957; doi:10.1042/CS20220472)
Supplement: Supplementary Figures S1-S3 and Tables S1-S2 [file CS-2022-0472_supp.pdf]

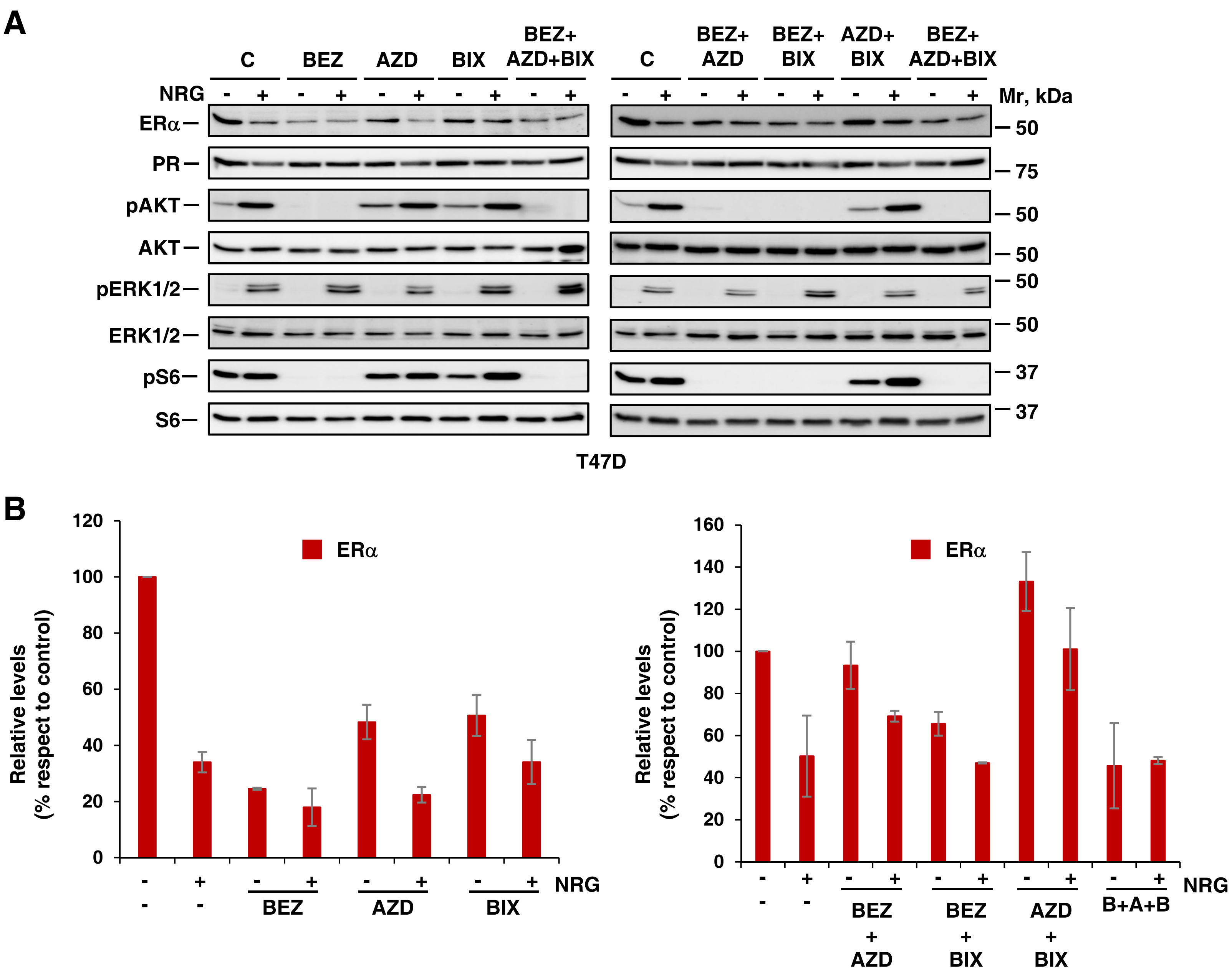
A
C
BEZ
AZD
BIX
BEZ+ AZD+BIX
BEZ+ AZD
BEZ+ BIX
AZD+ BIX
NRG
Mr, kDa
ERα
PR
pAKT
AKT
pERK1/2
ERK1/2
pS6
S6
50
75
37
T47D
B
ERα
Relative levels (% respect to control)
NRG
B+A+B

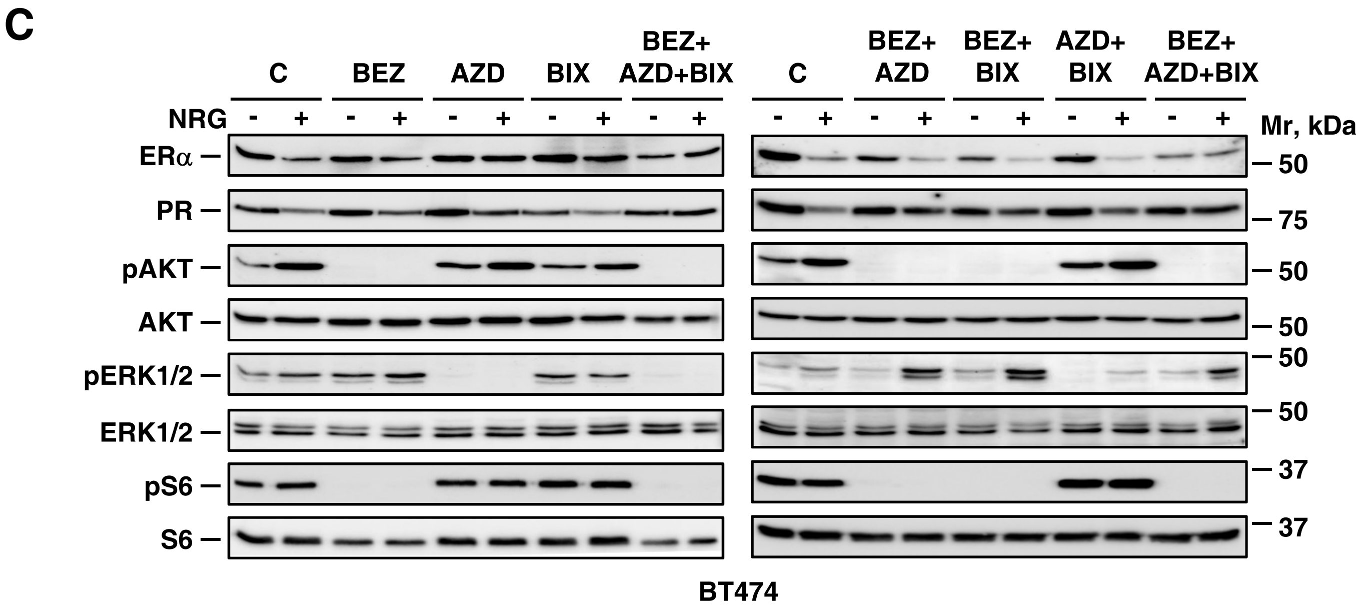
C
C
BEZ
AZD
BIX
BEZ+ AZD+BIX
C
BEZ+ AZD
BEZ+ BIX
AZD+ BIX
BEZ+ AZD+BIX
NRG
Mr, kDa
ERα
PR
pAKT
AKT
pERK1/2
ERK1/2
pS6
S6
50
75
50
50
50
50
37
37
BT474

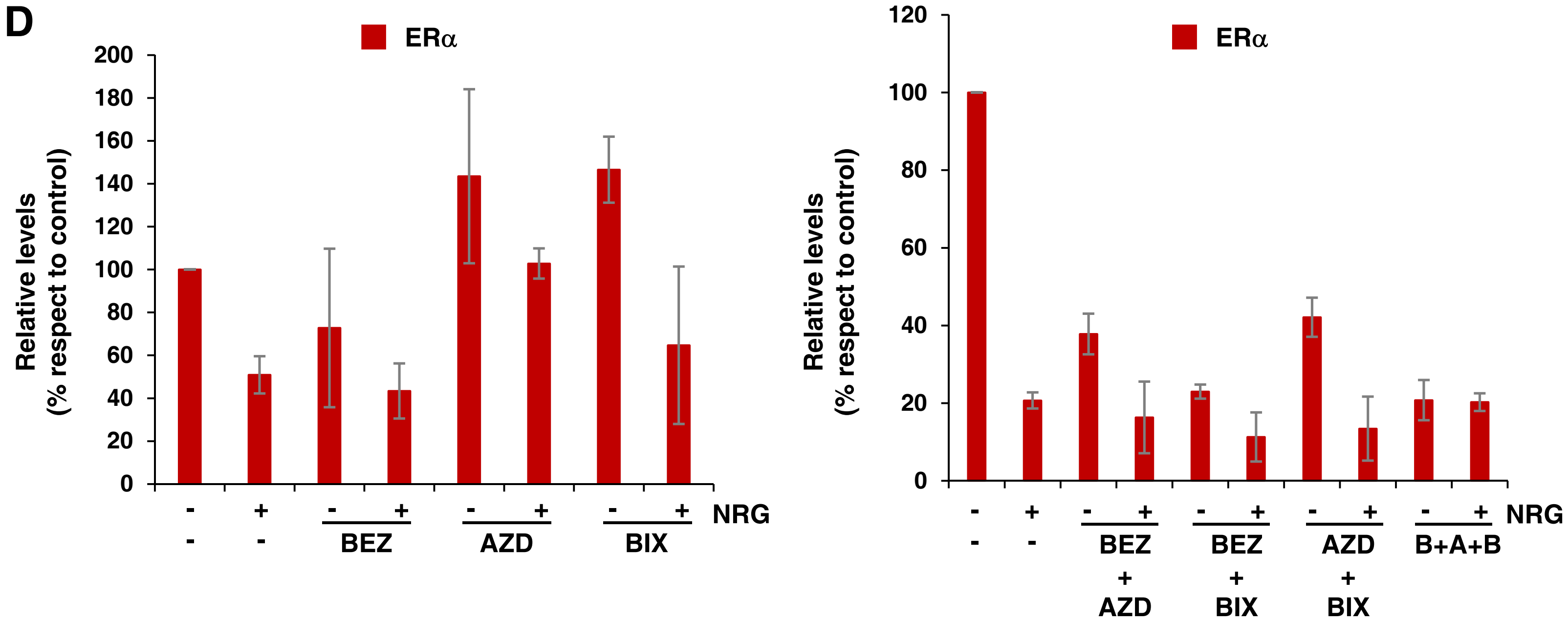
D
ERα
Relative levels (% respect to control)
0
20
40
60
80
100
120
140
160
180
200
NRG
BEZ
AZD
BIX
ERα
Relative levels (% respect to control)
0
20
40
60
80
100
120
NRG
BEZ + AZD
BEZ + BIX
AZD + BIX
B+A+B

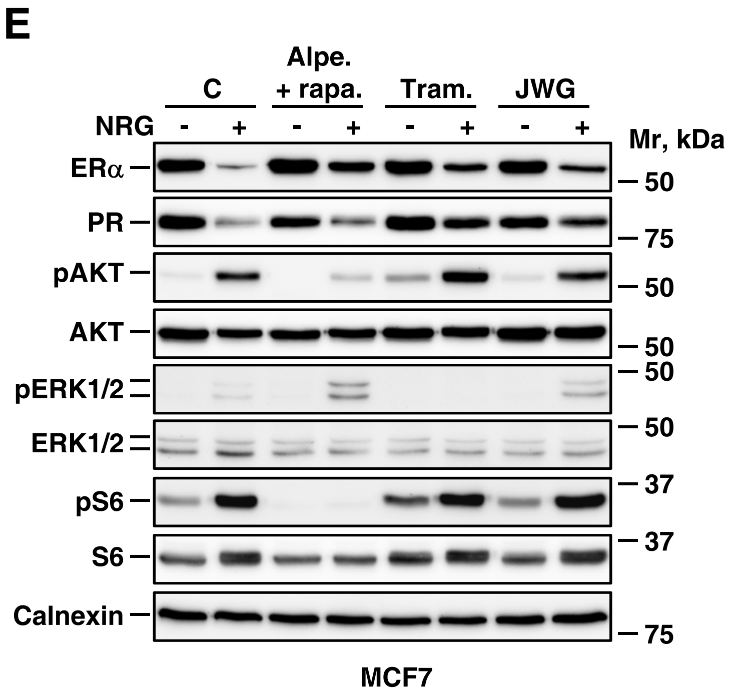

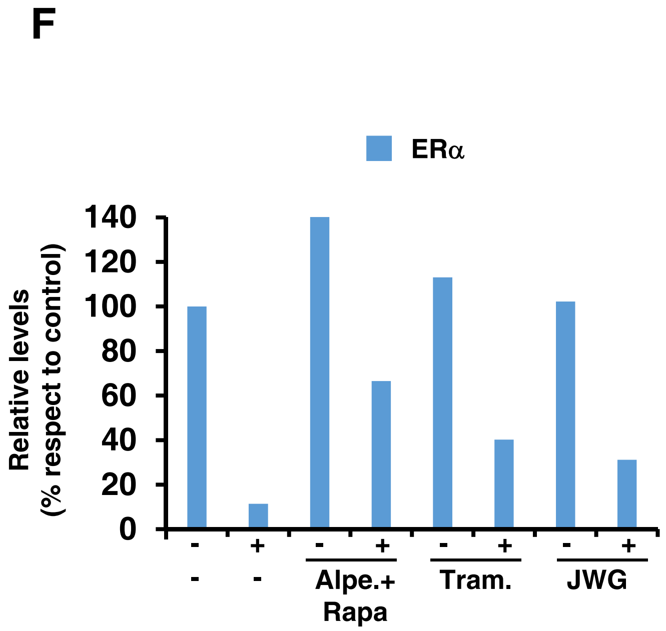

Supplementary Figure 1. T47D (A) and BT474 (C) cells were pretreated with a PI3K inhibitor (BEZ235, 1 μM), a MEK1/2 inhibitor (AZD6244, 5 μM), a MEK5 inhibitor (BIX02189, 10 μM) and with double and triple combinations of these inhibitors for 2 hours. Later, they were then stimulated with NRG for 24 hours. The expression levels of ERα, PR and several of the downstream proteins involved in NRG receptor signaling were performed by Western blot. (B and D) The graphics represent the quantitation of ERα levels corresponding to the mean ± SD of data from two independent experiments as performed in A and C. (E) MCF7 cells were pretreated with a PI3Kα inhibitor (Alpelisib, 1 μM) plus an mTOR inhibitor (Rapamycin, 100 nM), a MEK1/2 inhibitor (Trametinib, 100 nM) and an ERK5 inhibitor (JWG071, 5 μM) for 2 hours. Later, they were then stimulated with NRG for 24 hours. The expression levels of ERα, PR and several of the downstream proteins involved in NRG receptor signaling were performed by Western blot. (F) The graphics represents the quantitation of ERα levels corresponding to the experiment performed in E.

Supplementary Figure 1

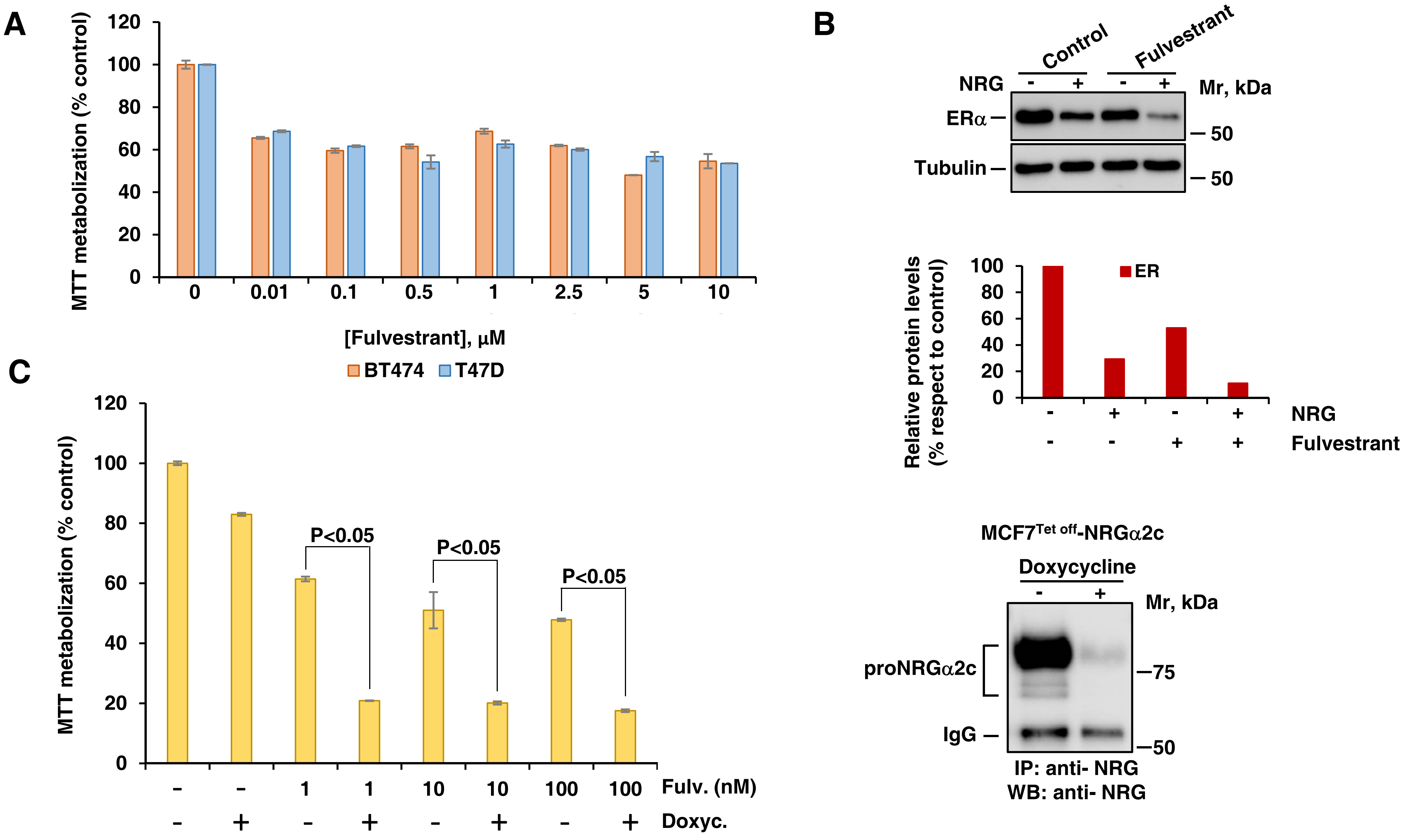

Supplementary Figure 2. (A) Dose-response analyses of the effect of fulvestrant on BT474 and T47D cells. Cells were treated with fulvestrant at the indicated doses and cell proliferation was determined by MTT metabolization 4 days later. The data are plotted as the percentage of MTT metabolization with respect to control. Results are shown as the mean ± SD of quadruplicates of an experiment repeated three times. (B) Levels of expression of ERα in MCF7 cells treated with NRG (10 nM) plus fulvestrant (1 μM) for 48 hours. The graphic represents the quantitation of the ERα levels corresponding to the upper panel. (C) Effect of the expression of NRG in MCF7^{TetOff}-NRGα2c cells treated with fulvestrant. MCF7^{TetOff}-NRGα2c cells were treated with doxycycline (10 nM) for 72 hours, and later the cells were treated with the indicated doses of fulvestrant. Cell proliferation was determined by MTT metabolization 4 days later. The data are plotted as the percentage of MTT metabolization with respect to control. Results are shown as the mean ± SD of duplicates of an experiment repeated two times. The expression of proNRGα2c in MCF7^{TetOff}-NRGα2c cells treated with doxycycline was analyzed by Western blot.

Supplementary Figure 2

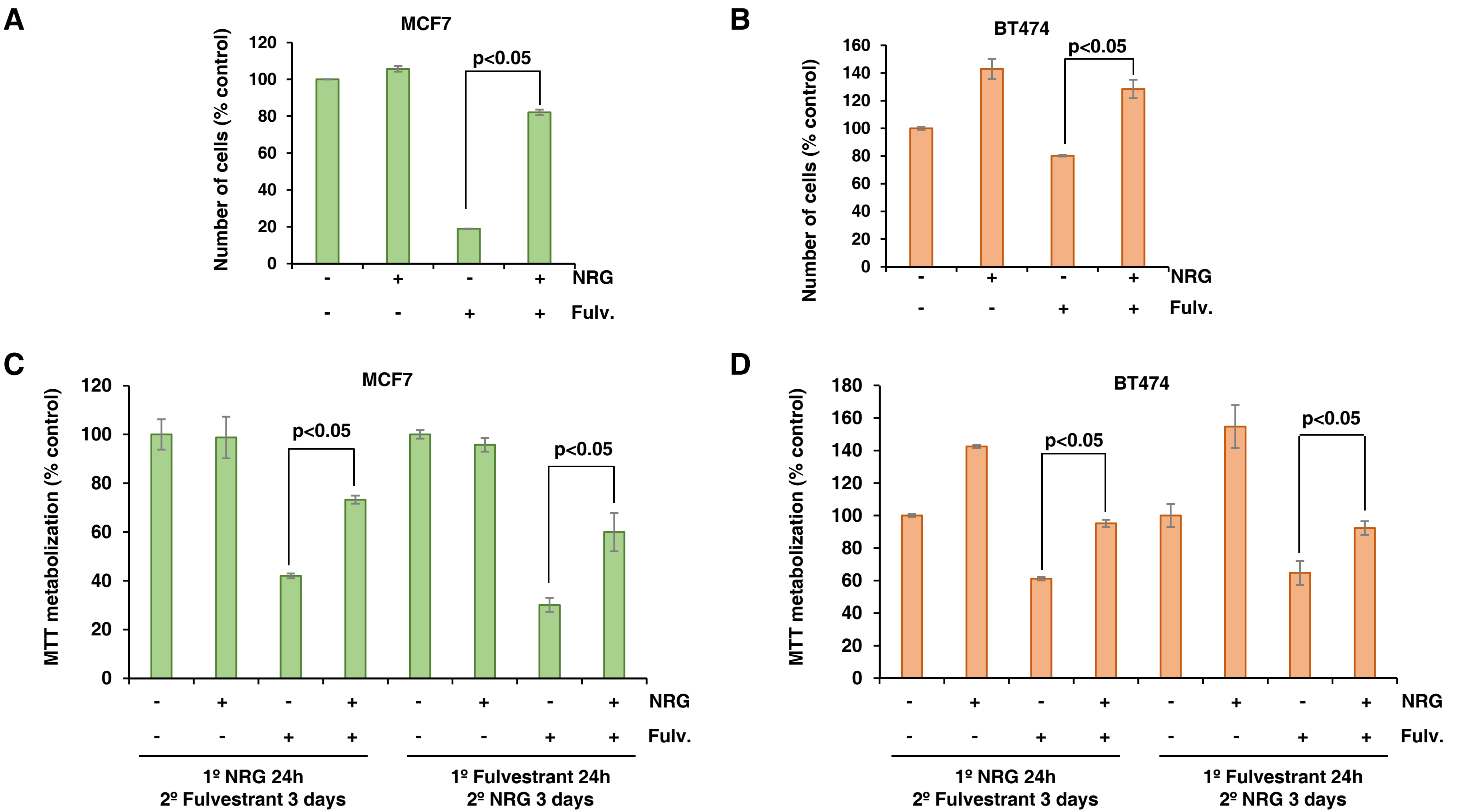

Supplementary Figure 3. (A-B) Effect of NRG in MCF7 or BT474 cells treated with fulvestrant. MCF7 (A) or BT474 (B) cells were stimulated with NRG (10 nM) for 24 hours, and later the cells were treated with fulvestrant (1 µM). Cell proliferation was determined by cell counting 4 days later. The data are plotted as the percentage of the number of cells respect to control. Results are shown as the mean ± SD of triplicates of an experiment repeated two times. (C-D) Effect of NRG in MCF7 or BT474 cells treated with fulvestrant. MCF7 (C) or BT474 (D) cells were stimulated and treated like experiments performed in A and B. Moreover, this experiment was also carried out in the reverse way, (pre-treating 24 hours with fulvestrant, and later stimulating with NRG for 4 days). Cell proliferation was determined by MTT metabolization. The data are plotted as the percentage of MTT metabolization with respect to control. Results are shown as the mean ± SD of quadruplicates of an experiment repeated two times.

Supplementary figure 3

A

	ESR1	*PGR*
	Mann-Whitney U **(p-value)**	***Mann-Whitney U*** **(p-value)**
**C vs. 1h**	<0.05	<0.05
**C vs. 3h**	<0.05	<0.05
**C vs. 6h**	<0.05	<0.05
**C vs. 12h**	<0.05	<0.05
**C vs. 24h**	<0.05	<0.05
**C vs. 48h**	<0.05	>0.05

MCF7

B

	ESR1	*PGR*
	Mann-Whitney U **(p-value)**	***Mann-Whitney U*** **(p-value)**
**C vs. 1h**	>0.05	>0.05
**C vs. 3h**	<0.05	>0.05
**C vs. 6h**	<0.05	<0.05
**C vs. 12h**	<0.05	<0.05
**C vs. 24h**	<0.05	<0.05
**C vs. 48h**	<0.05	<0.05

T47D

C

	ESR1	*PGR*
	Mann-Whitney U **(p-value)**	***Mann-Whitney U*** **(p-value)**
**C vs. 1h**	>0.05	>0.05
**C vs. 3h**	<0.05	<0.05
**C vs. 6h**	>0.05	<0.05
**C vs. 12h**	<0.05	<0.05
**C vs. 24h**	<0.05	<0.05
**C vs. 48h**	<0.05	<0.05

BT474

Supplementary Table 1

A

	ESR1 Mann-Whitney U (p-value)	*PGR* Mann-Whitney U (p-value)
**C (N) vs. BEZ (N)**	<0.05	>0.05
**C (N) vs. AZD (N)**	<0.05	>0.05
**C (N) vs. BIX (N)**	<0.05	>0.05
**C (N) vs. B+A+B (N)**	<0.05	>0.05

MCF7

B

	ESR1 Mann-Whitney U (p-value)	*PGR* Mann-Whitney U (p-value)
**C (N) vs. BEZ (N)**	<0.05	>0.05
**C (N) vs. AZD (N)**	>0.05	>0.05
**C (N) vs. BIX (N)**	>0.05	>0.05
**C (N) vs. B+A+B (N)**	>0.05	>0.05

T47D

C

	ESR1 Mann-Whitney U (p-value)	*PGR* Mann-Whitney U (p-value)
**C (N) vs. BEZ (N)**	<0.05	>0.05
**C (N) vs. AZD (N)**	>0.05	>0.05
**C (N) vs. BIX (N)**	>0.05	>0.05
**C (N) vs. B+A+B (N)**	<0.05	<0.05

BT474

Supplementary Table 2
